# Supplementary material for: Effects of Temperature and Salt Stress on the Expression of delta-12 Fatty Acid Desaturase Genes and Fatty Acid Compositions in Safflower
Source: Int J Mol Sci. 2023 Feb 1;24(3):2765. doi: 10.3390/ijms24032765 (PMC9917387; doi:10.3390/ijms24032765)
Supplement: Supplementary file 1 [file ijms-24-02765-s001.zip › Table S1.pdf]

Supplemental Table S1. Oligonucleotide primer names (Oligo), sequences and function in the amplification of Δ12 fatty acid desaturase genes in safflower.

Primer name	Nucleotide sequence (5′-3′)	Function/gene name
PF1	ATGGCTTGCAGACTCGCAGATT	Sequence clone (*CtFAD6*)
PR1	TTAAGCATAGTCGGGCATTACC	Sequence clone (*CtFAD6*)
PF2	TTCGTCCTCTACTACCTTGCC	Real-time PCR (*CtFAD2-1*)
PR2	CGCCGATGACTGTATTTCC	Real-time PCR (*CtFAD2-1*)
PF3	TTCCACAACATCACCGACAC	Real-time PCR (*CtFAD2-2*)
PR3	TCCTTCACCTCCTCATCTTTATC	Real-time PCR (*CtFAD2-2*)
PF4	AAGCCCAACAAACAAACCAT	Real-time PCR (*CtFAD2-10*)
PR4	CACCCTTGAACGATCCAGTAA	Real-time PCR (*CtFAD2-10*)
PF5	GGGAGCAGGTGGTCGGATGT	Real-time PCR (*CtFAD2-11*)
PR5	CGCCAGTGGAGTAGGAAGTTGAG	Real-time PCR (*CtFAD2-11*)
PF6	GTGGTGGGCATCCATCTTGTT	Real-time PCR (*CtEF1α*)
PR6	TACCTCCCAGGCTGATTGTG	Real-time PCR (*CtEF1α*)
PF7	GAGATGGCACCGTGAGTTATG	Real-time PCR (*CtUBCE2*)
PR7	GCCCTTCATGTACAGAGTTGTG	Real-time PCR (*CtUBCE2*)
PF8	cttggtaccgagctcggatccATGGCTTGCAGACTCGCAGATT	Sequence clone (*CtFAD6*)
PR8	tagatgcatgctcgagcggccgcTTAAGCATAGTCGGGCATTACC	Sequence clone (*CtFAD6*)
