# Supplementary figures and images for: Effects of Temperature and Salt Stress on the Expression of delta-12 Fatty Acid Desaturase Genes and Fatty Acid Compositions in Safflower
Source: Int J Mol Sci. 2023 Feb 1;24(3):2765. doi: 10.3390/ijms24032765 (PMC9917387; doi:10.3390/ijms24032765)

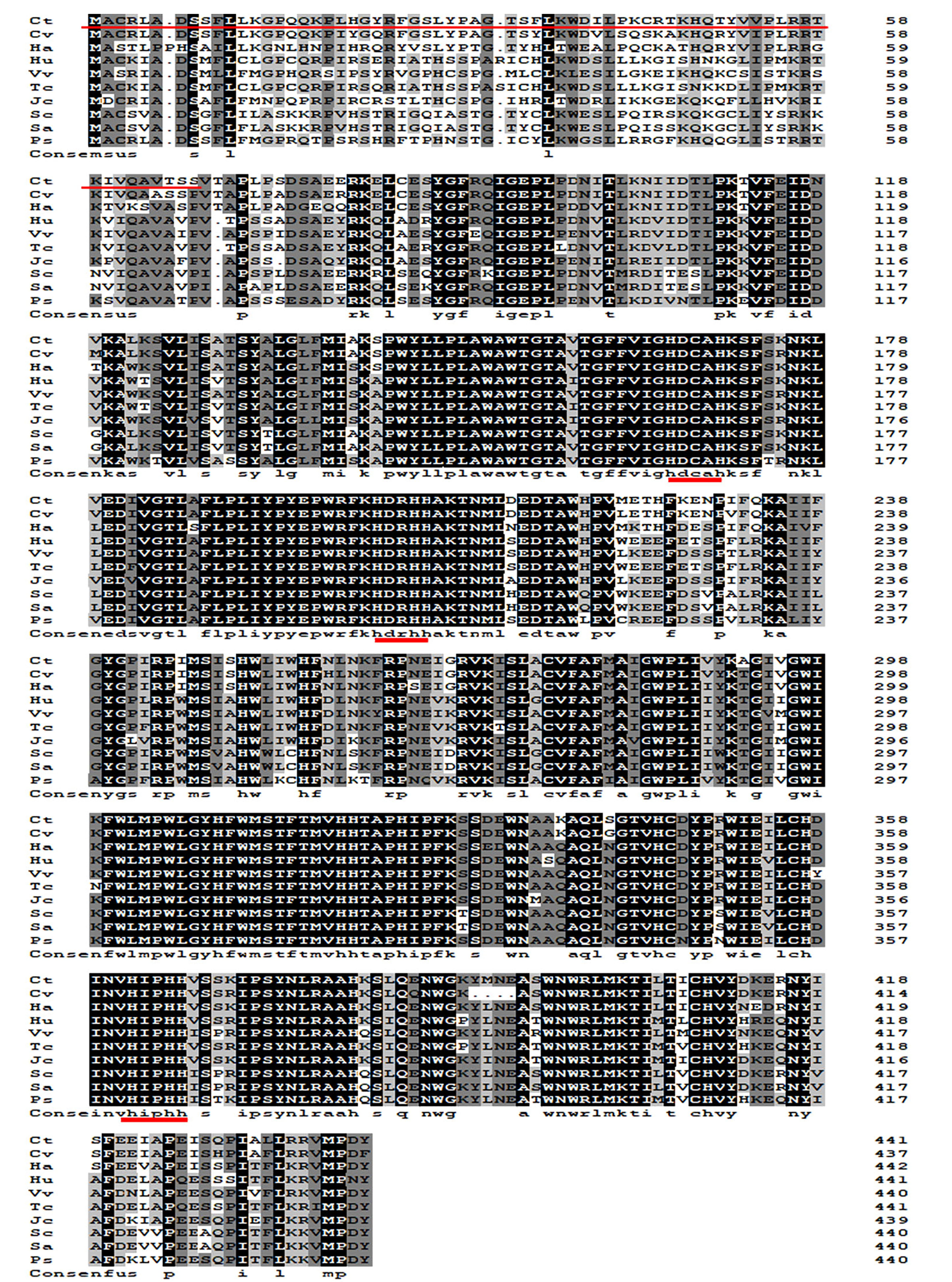

Supplement: Supplementary file 1 [file ijms-24-02765-s001.zip › Figure S1.jpg]
